# Lymphomatoid granulomatosis masquerading as interstitial pneumonia in a 66-year-old man: a case report and review of literature

**DOI:** 10.1186/1756-8722-2-39

**Published:** 2009-09-04

**Authors:** Ashima Makol, Kalyan Kosuri, Deimante Tamkus, Wanderley de M Calaca, Howard T Chang

**Affiliations:** 1 Department of Internal Medicine, Michigan State University, East Lansing, MI, USA; 2 Division of Pulmonary & Critical Care, Department of Internal Medicine, Wayne State University, Detroit, MI, USA; 3 Division of Hematology/Oncology, Michigan State University, East Lansing, MI, USA; 4 Department of Pathology, Sparrow Health System, Lansing, MI, USA; 5 Department of Neurology and Ophthalmology, Michigan State University, East Lansing, MI, USA

## Abstract

Lymphomatoid granulomatosis (LG) is a rare, Epstein-Barr virus (EBV)-associated systemic angiodestructive lymphoproliferative disorder that may progress to a diffuse large B cell lymphoma. Pulmonary involvement may mimic other more common lung pathologies including pneumonias. Therapeutic standards have not been established for LG, but rituximab, interferon-α2b (INF-α2b), and chemotherapy have shown to improve symptoms and long term prognosis.

We report a case of rapid respiratory deterioration in a 66-year-old man with clinical presentation, chest radiography, pulmonary function testing and high resolution computed tomography (HRCT) findings consistent with idiopathic interstitial pneumonia, but very poor response to antibiotics and low dose steroids. Lung biopsy showed histopathology consistent with LG that was confirmed by a positive in situ hybridization for Epstein - Barr virus encoded RNA (EBER). The patient was treated with rituximab and combination chemotherapy and showed significant initial clinical improvement with gradual resolution of abnormal findings on imaging. However, the patient developed pancytopenia as a complication of chemotherapy and died secondary to septic shock and renal failure that were refractory to medical management. Autopsy showed diffuse alveolar damage but no evidence of any residual LG within the lungs.

This case demonstrates that an open lung biopsy or video-assisted thoracoscopic surgical (VATS) biopsy is often necessary to rule out the presence of LG in order to determine the appropriate therapeutic strategy early in the course of illness to improve prognosis.

## Background

Lymphomatoid granulomatosis (LG) was first described as a clinicopathological entity in 1972 by Liebow et al [1]. It is a rare, angiocentric and angiodestructive, Epstein-Barr virus (EBV)-driven, T cell rich- B cell lymphoproliferative disorder (LPD) with clinical presentation varying widely from an indolent process to an aggressive B cell lymphoma. It usually presents in the fifth-sixth decade of life and is often associated with immunosuppression or immunodeficiency states. Men are affected twice as often as women (2:1) [2].

Lungs are most commonly involved, with less frequent involvement of skin, kidney, liver and central nervous system (Table 1) [1,3]. Pulmonary LG may present with cough, dyspnea or chest pain, and constitutional symptoms of fever and weight loss are common [4]. Less than 5% are asymptomatic but delay in diagnosis is common. Braham et al reported a patient whose presentation mimicked interstitial lung disease clinically [5]. Our case represents another presentation of LG masquerading as interstitial pneumonitis clinically.

**Table 1:** Clinical presentation of Lymphomatoid Granulomatosis-a review of literature

**Organ System Involved**	**Clinical Features**	**Diagnosis**	**Treatment and Prognosis**
1. Pulmonary (lung and mediastinal lymph nodes)	-Dyspnea, Cough, Chest pain, Fatigue, Non-productive cough -Constitutional symptoms -rarely, asymptomatic -Underlying Immunodeficiency e.g. AIDS - may clinically mimic pneumonia or interstitial lung disease [5]	-Chest Radiograph-non specific Differential Diagnosis: Pseudolymphoma, Interstitial Pneumonia, Wegener's Granulomatosis, Sarcoidosis, Metastasis [8] -High Resolution CT chest-peribronchovascular distribution of nodules and coarse irregular opacities, small thin walled cysts, and conglomerating small nodules [8] - Gold standard-Histopathology and Immuno-histochemical staining with EBV RNA in situ hybridization.	Progresses to malignant lymphoma in 13-47% cases [3,8] Mortality ranges from 53-63.5% [3,8] Treatment modalities-combination chemotherapy, Rituximab, Interferon-α2b, Autologous stem cell transplantation [10-13]

2. Central Nervous System (in 20% cases) [14,15]	-Spastic Paraparesis -Gait disturbances -Neurogenic Bladder -Central Diabetes Insipidus -Peripheral neuropathy -Concomitant Pulmonary involvement	-Elevated soluble IL-2 receptor level (normal 167-497 U/ml) -CSF-elevated protein, lymphocytic pleocytosis -MRI-spotty high intensity lesions on T2 imaging and enhancement with gadolinium contrast -PET scan-increased uptake of FDG -Gold standard-Biopsy and immuno-histochemical staining studies, EBV RNA in situ hybridization.	No well established treatment. CNS involvement is a marker of poor prognosis. Whole brain irradiation, chemotherapy, stem cell transplantation tried without much efficacy. Rituximab monotherapy demonstrating efficacy [14].

3. Others-skin, liver, kidney, spleen, mesenteric lymph nodes, etc	-Rash, subcutaneous nodules, ulceration. Usually non tender but occasionally pruritic -Usually associated with pulmonary or CNS LG	Work up as above	Treatment is along lines of systemic LG.

Chest radiographs are usually non-specific. Pulmonary nodules of varying sizes ranging from 1 to 9 cm are the most common findings (80% cases), but hilar adenopathy, pleural effusion, pneumothorax, pneumomediastinum and abscesses [4] also have been reported. Studies containing large radiographic series often report presence of diffuse bilateral nodules in the lower and peripheral lung fields with mass-like opacities [1-3,6-8]. Bronchoscopic biopsy is positive in up to 27% cases, but definitive diagnosis requires tissue biopsy obtained by open lung biopsy or video assisted thoracoscopic surgery (VATS) (3). Gross pathology of the lung lesions may consist of multiple yellow-white spherical masses with central necrosis with a solid or granular cheesy appearance [2]. Histologically, it is characterized by large atypical CD20+ B cells in a polymorphous inflammatory milieu of small lymphocytes, plasma cells, histiocytes (with karyorrhectic debris), and numerous CD3+ T cells (quantitatively outnumbering the B cells) with the infiltrate centered around bronchovascular and perivascular regions [8]. Epithelioid granulomas and giant cells are almost always absent despite the name 'granulomatosis'. EBV is demonstrable in the large atypical B cells by in situ hybridization of EBV encoded RNA. LG has been classified into 3 histological grades depending on the number of atypical large EBV-infected cells [9]. Grade 3 LG lesions most closely resemble clinically and pathologically the more conventional forms of diffuse large B cell lymphoma (DLBCL) and are treated in a similar manner. Most patients have grade 1-2 LG lesions at presentation.

Outcomes are variable and correlate with the histological grade. About one third of grade 1 lesions and two-thirds of grade 2 lesions progress to lymphoma [9]. The course of LG tends to be fulminant with a median survival of 14 months and mortality of 65-90%, with death resulting from progressive pulmonary involvement, extrapulmonary disease (particularly neurological) and/or complications of therapy [6].

Due to its rarity, standard treatment has not been established but it is important to diagnose and intervene early because of rapid progression. Therapy has ranged from observation to treatment with steroids or aggressive chemotherapy in various case series [10]. Low grade lesions may be treated with steroids alone. In view of similarity to EBV associated post transplant lymphoma, Interferon-α2b has often been used to treat LG due to its antiviral, antiproliferative and immunomodulatory properties, with good response [11]. Grade 1 and 2 diseases are often treated by interferon-α2b in combination with a humanized monoclonal anti-CD20 antibody (rituximab) [10] while Grade 3 lesions are treated like high grade lymphomas with aggressive chemotherapy. Combination chemotherapy, usually, R-CHOP regimen (rituximab, cyclophosphamide, doxorubicin, vincristine, prednisone) is used but response rates are poor at this grade [12]. Autologous stem cell transplantation has also been reported to be successful in refractory cases but the clinical implications of this modality have not been reported in large studies [13].

## Case Presentation

A 66-year-old Caucasian man with no significant past medical history presented with flu-like symptoms, progressively worsening shortness of breath, difficulty in breathing (NYHA class III) and dry cough over the past 2 weeks prior to presentation. He denied any fever, chills, sputum production, orthopnea or paroxysmal nocturnal dyspnea, anorexia, weight loss, recent or past exposure to tuberculosis, sick contacts, pets, recent travel or past exposure to cigarette smoke (active or passive), asbestos, silica, coal dust or chemicals. He maintained an active lifestyle walking 5 miles three times a week without overt dyspnea. He had no prior history of connective tissue disease or HIV and denied any history of skin rash or joint pains. Past surgical, social and family histories were non-contributory. He also had no history of prior use of any long term medications. He had presented to the hospital 4 days prior to present admission and a provisional diagnosis of community acquired pneumonia was made based on chest radiograph findings and he was discharged home on 2L of oxygen and Levofloxacin, but came back to the hospital due to lack of obvious improvement.

On examination, he appeared tired with shortness of breath. The temperature was 99.9°F, the pulse 124/min, respiratory rate 26/min and oxygen saturation of 86% on 2L. Skin was diaphoretic. There was no evidence of rash, pallor, lymphadenopathy, clubbing, joint swelling or edema. Auscultation of chest revealed diminished breath sounds with bilateral velcro-like fine inspiratory crackles. Laboratory studies showed a normal complete blood count, metabolic panel, liver function tests, cardiac enzymes, BNP, lactate, PT/INR, aPTT, fibrinogen and TSH. D-dimer was elevated at 15.4 (normal <1.6). ESR was 52 mm/hour. Rheumatoid factor was <5 (negative). ANA, p-ANCA and c-ANCA were negative. Blood cultures (bacterial/fungal/mycobacterial), urine legionella and streptococcal antigen were negative. Viral respiratory panel, including cytomegalovirus & Herpes Simplex Virus PCR were also negative. ABG showed hypoxemia with respiratory alkalosis. Pulmonary function tests showed restrictive pattern of lung disease. Transthoracic echocardiogram showed normal cardiac chamber sizes and left ventricular ejection fraction of 81%.

**Figure 1:**
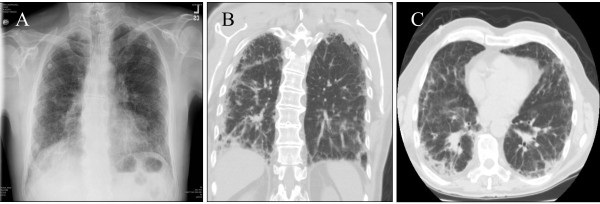
**A. Plain chest radiograph shows bibasilar infiltrates with a peripheral reticulonodular pattern superimposed on generalized interstitial changes involving the upper lobes and lung bases**. **B. (Coronal view) and C. (Axial View) **High Resolution Computed Tomography of the chest shows thickening of intralobular septa, septal line formation, parenchymal band formation and peribronchial thickening. Mild mediastinal lymphadenopathy is also noted.

Plain chest radiograph (Fig. 1A) showed bilateral basilar infiltrates and a peripheral reticulonodular pattern superimposed on generalized interstitial changes, involving the upper lobes as well as lung bases. High Resolution Computed Tomography (HRCT) of the chest (Fig. 1B and 1C) revealed moderate to severe thickening of intralobular septa, septal line formation, parenchymal band formation and peribronchial thickening, ground glass opacities and mild mediastinal lymphadenopathy (likely reactive, largest lymph node being 1.1 cm) was noted. This was consistent with idiopathic interstitial pneumonia (IIP) without a specific pattern. At this time, treatment with levofloxacin was continued and solumedrol was added for empirical therapy. Computed Tomogram (CT) of the abdomen/pelvis was normal with no evidence of retroperitoneal lymphadenopathy. Despite steroid therapy, the patient's respiratory status deteriorated over the next day requiring intubation and mechanical ventilation. Consequently, consent for wedge biopsy of the lung was obtained for a pathological diagnosis to guide further therapy.

**Figure 2:**
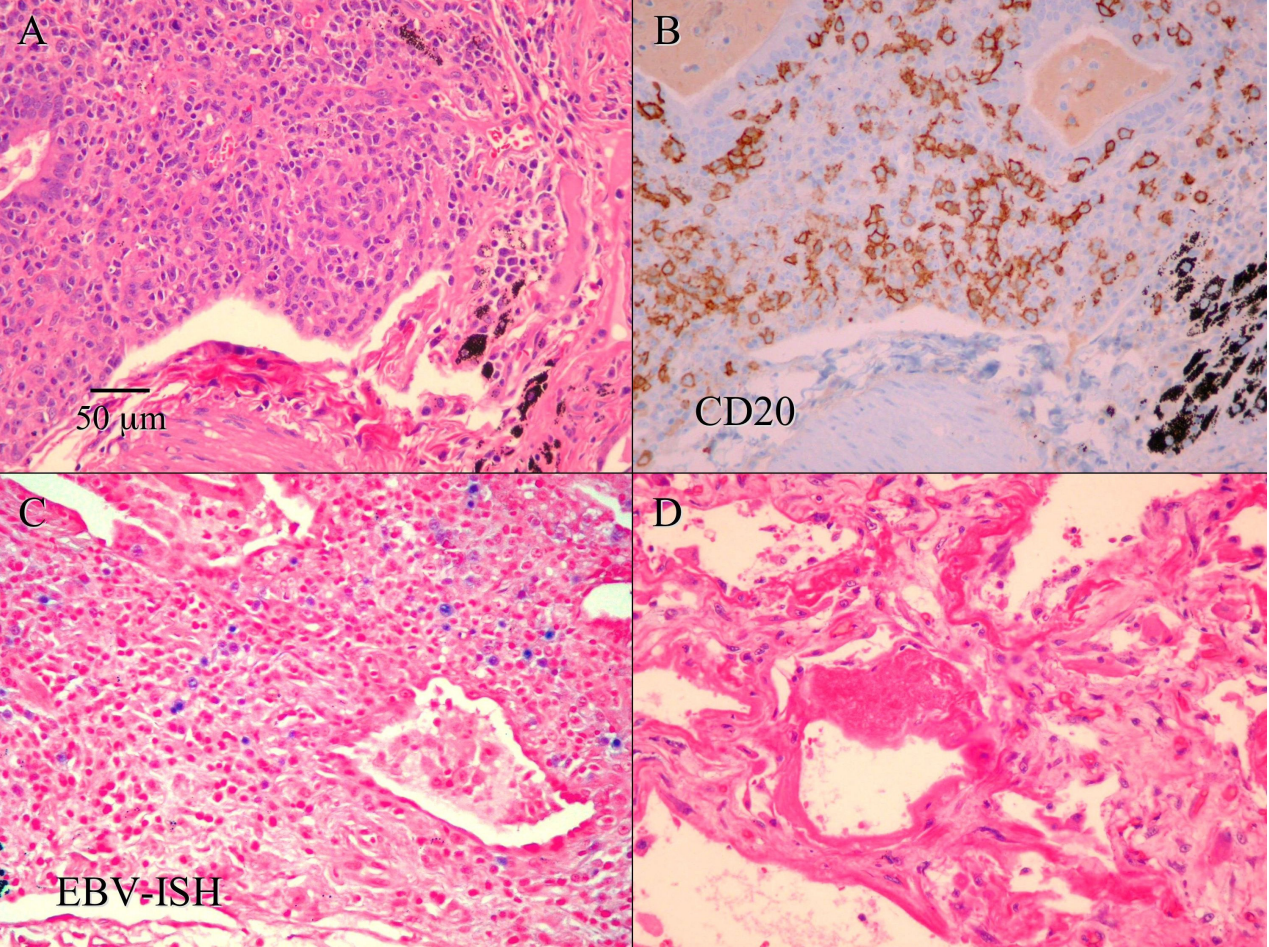
**A. Hematoxylin and eosin stain of the lung biopsy shows a polymorphic lymphoid infiltrate composed of large atypical cells, small lymphocytes and many plasma cells, with lymphoid cells infiltrating blood vessels and bronchial walls**. (Scale bar in A also applies to B-D, original magnification 200×) **B**. Immunohistochemistry on a section adjacent to A shows that that many large atypical cells are positive for CD20 (B cell marker). **C**. In situ hybridization for Epstein-Barr virus (EBV) encoded RNA (EBER) on a section adjacent to A shows that many large lymphocytes are positive for EBER (stained blue). **D**. A chest-only autopsy revealed diffuse alveolar damage in both lungs, with areas of edema, fibrin deposition, and hyaline membrane formation. There is no evidence of residual lymphomatoid granulomatosis.

A right lung wedge biopsy was obtained by video-assisted thoracoscopic surgery (VATS). A polymorphic lymphoid infiltrate composed of large atypical cells, small lymphocytes and many plasma cells was noted, with lymphoid cells infiltrating blood vessels and bronchial walls (Fig. 2A). Multinucleated large Reed Sternberg-like cells were also present along with foci of necrosis. Bronchial washings showed atypical benign bronchial cells and pulmonary macrophages with a few rare atypical Reed-Sternberg-like cells. Bone marrow biopsy was normal.

Immuno-histochemical studies of lung tissue showed predominance of T cells expressing CD3, CD5 and CD43 with a smaller population of large atypical cells expressing CD20 and CD79a (B cell markers) (Fig. 2B). Many CD138+ plasma cells exhibiting polyclonal staining pattern for kappa and lambda immunoglobulin light chains were also seen. Bone marrow biopsy revealed normal cellularity with no evidence of lymphoma. In situ hybridization for EBV encoded RNA (EBER) was positive within scattered large lymphoid cells throughout the biopsy specimen (Fig. 2C).

Based on the pathological and immuno-histochemical findings, a diagnosis of Lymphomatoid Granulomatosis was made. Treatment with high dose steroids and rituximab showed significant clinical improvement and he was extubated 4 days after starting therapy. Treatment was continued with cyclophosphamide, vincristine, doxorubicin and prednisolone chemotherapy and he showed gradual but slow resolution of clinical and x-ray findings. However, he subsequently developed pancytopenia consequent to chemotherapy, septic shock requiring increasing doses of pressors and acute renal failure which did not respond to aggressive management and he was terminally weaned per family wishes 4 weeks later. A chest-only autopsy revealed diffuse alveolar damage, likely secondary to sepsis or chemotherapy or both, but no evidence of residual LG was noted within the lungs (Fig. 2D).

## Discussion and Conclusion

This case illustrates that LG can clinically and radiographically mimic idiopathic interstitial pneumonia on presentation. However, rapid respiratory deterioration, without any other obvious etiology as in our patient, must prompt physicians to consider additional differential diagnoses. Although HRCT is an excellent modality in diagnosing interstitial pathology, we must be aware of potential mimics and proceed with open or VATS biopsy to obtain a pathological diagnosis in all patients who do not respond to empirical therapy. Early diagnosis and aggressive intervention, with interferon therapy, rituximab and chemotherapy in high grade LG can be life-saving for a patient with this rare yet treatable disease.

## Abbreviations

LG: Lymphomatoid granulomatosis; LPD: Lymphoproliferative disorder; HRCT: High Resolution Computed Tomography; VATS: Video Assisted Thoracoscopic surgery; EBV: Epstein-Barr Virus; EBER: Epstein-Barr Virus encoded RNA; R-CHOP: Rituximab, cyclophosphamide, doxorubicin, vincristine, prednisone combination chemotherapy; DLBCL: Diffuse large B cell lymphoma; IIP: Idiopathic Interstitial Pneumonia.

## Competing interests

The authors declare that they have no competing interests.

## Authors' contributions

AM was involved in conception and writing the manuscript. AM and KK participated in collection of clinical data and writing the manuscript. WDC made the pathological diagnosis on the biopsy and performed the autopsy. DT was the treating oncologist. HTC was involved in reviewing the pathology, preparing figures, and critical appraisal of the manuscript. All authors read and approved the final manuscript.

## Authors' Information

AM is an Internal Medicine Resident at Michigan State University. KK is a Pulmonary and Critical Care fellow at Wayne State University. DT is a hematology/oncology professor at Michigan State University. WC and HTC are pathologists in the Department of Pathology at Sparrow Health System, and HTC and DT are also associate professors at the Michigan State University.

## Consent

Written informed consent was obtained from the patient's next-of-kin for publication of this case report and accompanying images. A copy of the written consent is available for review by the Editor-in-Chief of this journal.

## References

[B1] Liebow AA, Carrington CR, Friedman PJ (1972). Lymphomatoid granulomatosis. Hum Pathol.

[B2] Jaffe ES, Wilson WH, Jaffe ES, Harris NL, Stein H, Vardiman JW (2001). Lymphomatoid granulomatosis. World Health Organization Classification of tumors Pathology and Genetics of Haemotopoietic and Lymphoid Tissues.

[B3] Katzenstein ALA, Carrington CB, Liebow AA (1979). Lymphomatoid granulomatosis: A clinicopathological study of 152 cases. Cancer.

[B4] McCloskey M, Catherwood M, McManus D, Todd G, Cuthbert R (2004). A case of Lymphomatoid granulomatosis masquerading a lung abscess. Thorax.

[B5] Braham E, Ayadi-Kaddour A, Smati B, Ben Mrad S, Besbes M, El Mezni F (2008). Lymphomatoid granulomatosis mimicking interstitial lung disease. Respirology.

[B6] Sheehy N, Bird B, O'Briain DS, Daly P, Wilson G (2004). Synchronous regression and progression of pulmonary nodules on chest CT in untreated lymphomatoid granulomatosis. Clin Radiol.

[B7] Guinee D, Jaffe E, Kingma D, Wallberg K, Krishnan J (1994). Pulmonary lymphomatoid granulomatosis. Evidence for a proliferation of Epstein Barr virus infected B-lymphocytes with a predominant T-cell component and vasculitis. Am J Surg Pathol.

[B8] Lee JS, Tuder R, Lynch DA (2000). Lymphomatoid granulomatosis: radiological features and pathologic correlations. AJR.

[B9] Jaffe ES, Wilson WH (1997). Lymphomatoid granulomatosis: Pathogenesis, pathology and clinical implications. Cancer Surv.

[B10] Jordan K, Grothey A, Grothe W, Kegel T, Wolf H-H (2005). Successful treatment of mediastinal Lymphomatoid granulomatosis with Rituximab monotherapy. Eur J Haemotol.

[B11] Wilson WH, Kingma DW, Raffeld M, Wittes RE, Jaffe ES (1996). Association of Lymphomatoid granulomatosis with Espstein-Barr Viral Infection of B lymphocytes and Response to Interferon-α2b. Blood.

[B12] Pisani RJ, DeRemee RA (1990). Clinical implications of the histopathological diagnosis of pulmonary Lymphomatoid granulomatosis. Mayo Clin Proc.

[B13] Lemieux J, Bernier V, Martel N, Delage R (2002). Autologous hematopoietic stem cell transplantation for refractory Lymphomatoid granulomatosis. Hematology.

[B14] Ishiura H, Morikawa M, Hamada M, Watanabe T, Kako S (2008). Lymphomatoid Granulomatosis Involving Central Nervous System Successfully treated With Rituximab alone. Arch Neurol.

[B15] Mizuno T, Takanashi Y, Onodera H, Shigeta M, Tanaka N, Yuya H (2003). A case of Lymphomatoid granulomatosis/angocentric immunoproliferative leson with long clinical course and diffuse brain involvement. J Neurol Sci.

